# Medical thoracoscopy for exudative pleural effusion: an eight-year experience from a country with a young population

**DOI:** 10.1186/s12890-017-0499-y

**Published:** 2017-11-22

**Authors:** Merlin Thomas, Wanis H. Ibrahim, Tasleem Raza, Kamran Mushtaq, Adeel Arshad, Mushtaq Ahmed, Salma Taha, Saber Al Sarafandi, Hanfa Karim, Hisham A. Abdul-sattar

**Affiliations:** 1 Pulmonary Division, Department of Medicine, Hamad General Hospital, P.O.BOX 3050 Doha, Qatar; 2 Department of Medicine, Hamad General Hospital, P.O.BOX 3050 Doha, Qatar

**Keywords:** Medical thoracoscopy, Pleural effusion, Tuberculosis, Malignancy

## Abstract

**Background:**

With the exception of areas with high prevalence of tuberculosis, medical thoracoscopy is becoming the diagnostic modality of choice for exudative pleural effusions. The aims of this study were to determine the diagnostic yield and safety of medical thoracoscopy for exudative pleural effusions and ascertain the etiology of such effusions in Qatar.

**Methods:**

This is a retrospective-descriptive study of 407 patients who underwent diagnostic medical thoracoscopy for exudative pleural effusions from January, 2008 till December, 2015 at the only tertiary referral center performing this procedure in Qatar.

**Results:**

Tuberculosis was the most common etiology of exudative pleural effusions in Qatar accounting for 84.5% of all causes. Around 85% of patients were young males (mean age of 33 ± 12.1 years). The diagnostic yield of medical thoracoscopy for tuberculous pleural effusion was 91.4%. Malignant pleural effusions accounted for 5.2% of cases. Minor bleeding occurred in 1.2% of cases with no procedure-related mortality observed.

**Conclusion:**

Medical thoracoscopy is a very safe procedure. Tuberculous pleuritis is by far the most common etiology of exudative pleural effusions in Qatar. Closed needle biopsy is a worth consideration as an initial safe, easy and low-cost diagnostic modality for exudative pleural effusions in this country.

## Background

The first thoracoscopy (also termed pleuroscopy) was introduced by Hans-Christian JacÖbaeus in 1910 for replacement of pleural fluid with air as a treatment of tuberculous pleural effusion [1, 2]. In the 1990s video-assisted surgical thoracoscopy (VATS), which is performed under general anesthesia, was introduced. Medical thoracoscopy (MT) (a less invasive and less expensive procedure with a single port of entry) can be performed under local anesthesia and conscious sedation, usually by pulmonologists. MT is primarily a diagnostic procedure but can also be applied for therapeutic purposes such as pleurodesis and removal of pleural adhesions [2]. Qatar is an oil and gas-rich nation with a total population of 2,437,790 (2015 estimate). People below the age of 45 years constitute around 85% of the population of Qatar [3] and the country relies heavily on this labor force for its economic and industrial projects. The country is currently witnessing comprehensive reforms in its health care system aiming at achieving a world-class, patient-centered health care system by 2030. Hospitals in Qatar are of high standards and all accredited by the Joint Commission International. Exudative pleural effusions of unclear etiology generally require pleural biopsy for definite diagnosis. The etiology varies across different parts of the world. While pneumonia, malignancy, and thromboembolism account for most exudative effusions in the United States [4], tuberculosis (TB) is the most frequently encountered etiology in other developed as well as developing countries [5]. The objectives of the current study were to determine the yield and safety of MT as a diagnostic tool for exudative pleural effusions as well as ascertain the etiology of this type of effusion in a country with a predominantly young population.

## Methods

This was a retrospective-descriptive study of all patients who underwent MT to confirm the etiology of undiagnosed exudative pleural effusions and in whom initial hematological, microbiological, and cytological analyses were inconclusive. The diagnosis of exudative pleural effusion in the current study was based on Light’s criteria [4]. The study was conducted at Hamad general Hospital (the only tertiary referral center performing MT in the State of Qatar) during the period from January, 2008 to December, 2015. The study protocol was approved by the Medical Research Centre at Hamad Medical Corporation.

### Thoracoscopic procedure

MT at Hamad General Hospital is performed in the bronchoscopy suite by pulmonologists under local anesthesia and conscious sedation. A rigid thoracoscope (Karl Storz Endoskope; Karl Storz; Tuttlingen, Germany) is used for such procedure. The patient usually lies on the healthy side in a lateral decubitus position with the involved side up. An anterolateral thoracic approach in the 5th or 6th intercostal space is predominantly used with ultrasound guidance. Following a 1-2 cm incision, blunt dissection is done with an artery forceps and a trocar with an inner diameter of 8 mm is inserted through a cannula followed by insertion of thoracoscope. The pleural cavity is thoroughly inspected and around 2-4 biopsies are taken from the abnormal lesions on parietal pleura by a lateral “lift and peel” technique. Multiple biopsies are taken if no gross abnormalities are noted and the samples are sent for histopathology and culture for Mycobacterium tuberculosis. After thoracoscopy, a 24-French chest tube connected to an underwater sealed drain is routinely inserted for drainage of iatrogenic pneumothorax and the tube is removed once pneumothorax has resolved.

### Data collection

A structured data sheet in view of research study design and objectives was designed and created to collect all required data such as demographic details, pleural fluid characteristics, site of effusion, visual appearances on thoracoscopy, complications from the procedure, length of stay as well as pathological and microbiological diagnostic details. For quality assurance, all investigators who were involved in data collection received prior training on how to collect the data and complete the study data sheet.

### Statistical analysis

Categorical and continuous values were expressed as frequency (percentage) and mean ± SD or median. Descriptive statistics were used to summarize demographic and all other clinical characteristics of the study subjects. The primary outcome variable was to evaluate and assess the outcome and complications rate secondary to medical thoracoscopy and enlist causes of exudative effusions. Associations between two or more qualitative variables were assessed using the chi-square test or Fisher Exact test as appropriate. Relationship between two quantitative variables was examined using Pearson’s correlation coefficients. A 2-sided *P* value less than 0.05 was considered statistically significant. All statistical analyses were performed using SPSS 19.0 (SPSS, Inc., Chicago, Illinois).

## Results

The diagnostic accuracy of MT for tuberculous pleural effusion (TPE) was 91.4% with a sensitivity of 90% and specificity of 100%. The demonstration of one or more of following abnormalities in pleural biopsy specimen in a patient with high pretest clinical probability for TB was considered diagnostic of TPE: (I) presence of acid-fast bacilli on stain or culture; (II) caseating granulomas; (III) epithelioid cell granuloma with no evidence of other granulomatous diseases. Table 1 presents the demographic characteristics of the study subjects. Over a period of 8 years, a total of 407 patients underwent diagnostic MT for exudative pleural effusions. More than 85% of the patients were males with a mean age of 33 ± 12.1 years. There was a significant difference in the mean age group of patients with TPE (30.8 ± 9.5 years) and the Non TPE patients (46.8 ± 15.8 years) (*P* < 0.0001). Majority of patients who underwent diagnostic MT during the study period were young expatriates from Indian subcontinents and they constituted 77% of the TPE cases. Table 2 shows the etiology of pleural effusions in the study subjects. By far, tuberculous pleuritis was the most common etiology of exudative pleural effusions during the study period accounting for 84.5% of all causes. Sputum culture grew *Mycobacterium tuberculosis* in 65 (19%) patients with TPE. Most of the exudative pleural effusions in the current study were unilateral with right-sided effusions being more common than left-sided effusions (55 and 40% respectively). Bilateral effusions were seen in only 5% of patients. Forty five percent of effusions were moderate in size (between one-third and two-thirds of hemithorax), 22% were mild (less than one-third of hemithorax) and 19% were massive (more than two-third of hemithorax or causing midline shift). Parenchymal infiltrates were noted in 111 patients of whom 90 patients had TPE (Missing data in more than 50% of patients). Interestingly, malignant pleural effusions accounted for only 5.2% of the cases with metastatic pulmonary adenocarcinoma being the most commonly encountered neoplasm. The most common findings on gross thoracoscopic appearance of the pleura were hyperemia, sago-like nodules and multiple thin adhesions (around 51% of patients) (Table 3). Granulomatous inflammation was the most commonly encountered histologic finding (in around 75% of cases) followed by non-specific fibrinous pleuritis (17%) (Table 4). Table 5 presents the complications and post-operative care findings of MT. The median length of stay for patients who underwent MT was 3 days. Minor bleeding occurred in 1.2% of cases with no procedure-related mortality observed.

**Table 1:** Demographic characteristics of the study subjects

Characteristic	n (%)
Mean age	33.3 ± 12.1
Gender (*n* = 407)	
Male	357 (87.7)
Female	50 (12.3)
Ethnicity (*n* = 407)	
Indian Sub-continent	295 (72.5)
Philippines	29 (7.1)
Middle east	35 (8.6)
African	36 (8.8)
Others^a^	12 (3)
Occupation (*n* = 325)	
Unskilled	197 (48)
Semiskilled	76 (19)
Skilled	31 (8)
Others^b^	21 (5)

^a^Others - Iran, Thailand, Europe, Unknown

^b^Others - Housewife, retired, student

**Table 2:** Etiology of exudative effusions (*n* = 401)

Etiology	n (%)
Tuberculous pleural effusions	344 (84.5)
Para-pneumonic effusions	22 (5.4)
Malignant Effusions	21 (5.2)
Metastatic Pulmonary Adenocarcinoma	8 (38)
Metastatic Breast carcinoma	4 (19)
Lymphoma	4 (19)
Malignant mesothelioma	3 (14)
Malignant epithelial neoplasm	1 (5)
Squamous cell carcinoma Lung	1 (5)
Idiopathic	12 (2.9)
Others^a^	3 (0.7)

6 patients were lost to follow up

^a^Others –Inflammatory (2) Lymphangioleiomyomatosis (1)

**Table 3:** Gross thoarcoscopic findings (*n* = 390)

Findings	n (%)
Inflammation + sago like nodules ± adhesions	205 (51.2)
Inflammation +/− adhesions^a^	128 (32.4)
Inflammation with nodules/masses/plaques^a^	33 (8.3)
Normal	27 (6.9)

^a^Inflammation defined as edematous, deep and pink pleura

**Table 4:** Histological and microbiological findings (*n* = 407)

Pathology	n (%)
Granulomatous inflammation	304 (74.7%)
Fibrinous pleuritis	69 (17)
Malignant	13 (3.2)
Normal pleura	9 (2.2)
Others^a^	12 (2.9)

^a^Others Inadequate sample-11(2.7) Lymphangioleiomyomatosis (1)

**Table 5:** Post thoracoscopy care and complications (*n* = 407)

Characteristic	Value
Length of Stay	
Median	3 (IQR^a^ – 5)
Duration of chest tube	Days
Median	1 (IQR- 0)
Complications	n (%)
Minor Bleeding	5 (1.2)
Persistent Air leak >3 days	15 (3.7)

^a^ *IQR* Interquartile range

## Discussion

Among the most common respiratory illnesses encountered in day-to-day clinical practice are undiagnosed exudative pleural effusions. Thoracentesis is usually the first diagnostic procedure performed to identify the etiology of these effusions. Although etiologies vary from country to country, malignant diseases, tuberculosis and empyema remain the most commonly encountered causes for exudative pleural effusions. Unfortunately, 40% of patients with malignant pleural effusions remain undiagnosed after a routine cytological examination of the pleural fluid and TB culture is positive in only 20% of TPE [6, 7]. Blind pleural biopsy using Abrams needle is easy to perform, safe, and inexpensive. Although the value of closed needle biopsy in malignant pleural effusions is still a matter of controversy, it has a high yield (up to 80%) in TPE particularly when combined with pleural fluid TB culture. This has been attributed to the diffuse pattern in which TB affects the pleura. Hence, closed needle biopsy remains an important diagnostic tool in countries where the pre-test probability of TB is high and has been recommended as the initial diagnostic modality in these areas [8–12]. Despite being more expensive than blind pleural biopsy, MT is becoming the standard diagnostic modality for undiagnosed exudative pleural effusions in developed and some developing countries as it enables direct visualization of the pleural space. Different studies from many countries have confirmed the high yield of MT in diagnosing exudative pleural effusions that exceeds 70% and can reach as high as 95% in malignant effusions and 99% in TPE [9, 13–16]. Taking into consideration the high prevalence of TPE in the study subjects, the diagnostic yield of MT in the current study is lower than what was anticipated. One explanation for the relatively lower yield in Qatar is the low threshold of performing MT for cases with extensive pleural adhesions (due to non-visualization) which has resulted in the finding of non-specific fibrinous pleuritis in as high as 17% of cases (Table 4). Furthermore, the number of thoracoscopic biopsies (2-4 biopsies) performed at Hamad General Hospital per case is lower than the international recommendation and may be a contributor to the low diagnostic yield [12, 17]. Two striking findings have been observed in the current study. The first was the very high percentage of TPE (as compared to malignant effusions) diagnosed by MT in Qatar (84.5% vs. 5.4%). This finding has not been observed in previous studies from other countries including those with high TB prevalence [16, 18]. The other striking finding was the young age of patients with undiagnosed exudative pleural effusions who underwent MT. There are 3 plausible reasons for these findings; firstly, people below the age of 45 years constituted 85% of the total population in Qatar. Labor force from countries with high TB prevalence constitutes a significant proportion of such young population as well as 97% of TB cases encountered in this country [19]. In contrast to pleural TB in developed countries, Ibrahim, et al. in a previous study of pleural TB in Qatar found that younger age groups were the most commonly affected by the disease (84% are below the age of 45 years, with mean age of 31.5). Furthermore, the pleural involvement in most of the cases resulted from primary infection rather than reactivation of an old pulmonary TB [7]. Secondly, in contrast to other developing countries where closed needle biopsy is the initial diagnostic consideration in suspected pleural TB, this procedure is very rarely performed nowadays in Qatar and has largely been replaced by MT. Moreover, owing to the highly effective and strict National TB Control Program in Qatar, there is usually emphasis on tissue or microbiologic diagnosis of TB in the country. The median length of stay (LOS) and duration of chest tube in the current study were 3 days and 1 day respectively. These findings are in agreement with international figures [12]. Nevertheless, we believe that other factors (unrelated to the procedure) such as waiting for confirmatory histologic reports and the process of incorporating patient’s data in TB registry prior to discharge are important contributors to the LOS following MT in Qatar. Recently, it has become feasible in a number of countries to perform the procedure as a day-case [10]. The current study was the first to document the experience with MT in this country with a young population. The results of this study can be generalized to the whole country as Hamad General Hospital is the only tertiary referral center performing MT in Qatar and receives referrals from other health care facilities in the country. Nevertheless, in addition to the limitations inherent in a retrospective study, an important limitation of this study is the lack of comparison between MT and closed needle biopsy with regard to diagnostic yield, safety and cost effectiveness particularly in cases with suspected TPE from this region. Furthermore the finding of a high percentage of nonspecific fibrinous pleuritis due to extensive adhesions has limited the yield of MT in the current study.

## Conclusion

This study has confirmed the safety and value of MT as a diagnostic modality for undiagnosed exudative pleural effusions. In contrast to studies conducted in other parts of the world (including those with high TB prevalence), the current study confirmed that tuberculous pleuritis is by far the most common cause of undiagnosed exudative pleural effusion in Qatar. Furthermore, there is a very low threshold of using MT (rather than closed needle biopsy) for cases with suspected pleural TB. Owing to the high prevalence of TPE as compared to malignant effusions in Qatar and the previously reported high yield of closed needle biopsy, we recommend that closed needle biopsy rather than MT to be the initial safe, easy and low-cost diagnostic modality when TPE effusion is suspected.

## Abbreviations

LOS: Length of stay
MT: Medical thoracoscopy
TB: Tuberculosis
TPE: Tuberculous pleural effusion
